# Effects of saw palmetto fruit extract intake on improving urination issues in Japanese men: A randomized, double‐blind, parallel‐group, placebo‐controlled study

**DOI:** 10.1002/fsn3.1654

**Published:** 2020-06-17

**Authors:** Ikuya Ishii, Tatsuya Wada, Tsuyoshi Takara

**Affiliations:** ^1^ Quality Control Department YAWATA CORPORATION Tottori Japan; ^2^ NIHON PHARMACEUTICAL Co., Ltd Tokyo Japan; ^3^ Medical Corporation Seishinkai Takara Clinic Tokyo Japan

**Keywords:** IPSS, KHQ, Saw palmetto, sleep, subjective symptoms, urination issues

## Abstract

The aim of the present study was to investigate the effects of 12‐week consumption of saw palmetto fruit extract (320 mg per day) on urination issues. A total of 44 Japanese men aged 40–69 years who experienced urination issues and awaken ≥2 times at night to urinate participated in a randomized, double‐blind, placebo‐controlled study between June and December 2017. All subjects were randomly allocated into a saw palmetto fruit extract group (SP group, *n* = 22) or a placebo group (P group, *n* = 22) using a computerized random number generator. Each group took their assigned one capsule every day for 12 weeks. Subjective symptoms and impact on daily life were assessed using the international prostate symptom score (IPSS) as a primary outcome, King's health questionnaire (KHQ), and overactive bladder symptom score. A safety evaluation was also performed. A total of 20 subjects in each group were analyzed. There was a significant group–time interaction for total IPSS. The SP group also showed a significant decrease in IPSS at 8 weeks compared with the P group, suggesting reduced subjective symptoms related to urination issues. We observed no adverse effects. The consumption of saw palmetto fruit extract capsule for 12 weeks relieved subjective symptoms related to urination, which suggests improvement of the issue in healthy Japanese men.

## Graphical abstract

The consumption of saw palmetto fruit extract capsules for 12 weeks relieved urination‐related subjective symptoms, which suggests improved issues, such as urinary urgency, frequent urination, urinary incontinence, and awaken ≥2 times at night to urinate.

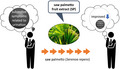

## 1 INTRODUCTION

Saw palmetto (*Serenoa repens*) is a palm tree, which grows throughout the southeastern part of North America, and its fruit has been used as traditional medicine for urological diseases (Sosnowska & Balslev, 2009). Saw palmetto fruit extract (SP) is approved as a drug to treat lower urinary tract symptoms (LUTS) in men with mild‐to‐moderate benign prostatic hypertrophy (BPH) in Europe (Committee on Herbal Medicinal Products 2016). Furthermore, SP is used as a dietary supplement ingredient in the United States and other countries, and various products containing SP are on the market in Japan, as well.

A previous study confirmed the effects of SP on urination in vivo and in vitro (The Consumer Affairs Agency 2012). Additionally, according to the Food Functionality Evaluation Model Project result report regulated by the Consumer Affairs Agency of Japan, an evaluation of SP was determined to alleviate mild‐to‐moderate BPH with symptoms such as the frequent urination and dysuria (The Consumer Affairs Agency 2012).

Saw palmetto fruit extract is obtained by either ethanol or supercritical fluid extraction, and its ingredients of fatty acids and sterols are approved by the European and US pharmacopoeia. However, the quality of SP depends on the procedure and material manufacturers. Most studies of SP by the Food Functionality Evaluation Model Project evaluated the extracts by ethanol; studies for the extracts by supercritical fluid were insufficient. Gerber GS et al confirmed the efficacy of saw palmetto extract for LUTS (Gerber, Kuznetsov, Johnson, & Burstein, 2001); the saw palmetto extract used by Gerber GS et al and the current report are manufactured by the same maker using the supercritical fluid extraction method.

Several studies of SP have provided sufficient evidence for subjects with slight‐to‐moderate BPH or with LUTS; however, few studies have been performed with healthy Japanese men (Najima, Munekata, & Nishikata, 2017; The Consumer Affairs Agency 2012).

Therefore, in this study, we investigated the effect of SP on healthy Japanese men experiencing urination issues without receiving medications or treatment. We used IPSS as the primary outcome in this study because Gerber GS et al showed that SP improved IPSS (Gerber et al., 2001).

## 2 MATERIALS AND METHODS

### 2.1 Design

The present study was randomized, double‐blind, placebo‐controlled, and the study protocol was approved by the independent ethical committee of the clinic set up by Medical Corporation Seishinkai on June 19, 2017 (Approval ID: 1706–1704‐YB02‐01‐TC). This study complied with the ethical principles of the Declaration of Helsinki (2013) and ethical guidelines for medical and health research involving human subjects in Japan, satisfying the requirements for medical ethics. The protocol was registered at the University Hospital Medical Information Network Clinical Trials Registry (UMIN000027903).

### 2.2 Subjects

The target subjects of this study were Japanese men aged 40–69 years who were experiencing urination issues, such as urinary urgency, increased urinary frequency, urinary incontinence, and awaken ≥2 times at night to urinate. The exclusion criteria were as follows: (a) medical history of a malignant tumor, heart failure, or myocardial infarction; (b) undergoing treatment for any of the following chronic diseases: arrhythmia, liver disease, kidney disease, cerebrovascular disease, rheumatism, diabetes mellitus, hyperlipidemia, hypertension, or any other chronic diseases; (c) undergoing treatment or medication for prostatic hypertrophy, overactive bladder (OAB), urolithiasis, and others; (d) daily consumption of “Foods for Specified Health Uses” or “Foods with Function Claims”; (e) regular use of medications, including herbal medicines or/and supplements; (f) allergies to medications and/or products related to the study substance; (g) participation in another clinical study within 3 months of signing the informed consent form for this study; and (h) considered inappropriate for this study by the primary investigator.

All subjects were recruited through a website operated by ORTHOMEDICO Inc. (Tokyo, Japan) between June and August 2017. All subjects were comprehensively informed of the study protocol and signed informed consent prior to participation in the study at ORTHOMEDICO Inc. office. No subject was affiliated with the sponsors or funders companies. The intervention period was between June and December 2017.

### 2.3 Sample size

According to a study by Cohen (1992), the effect size (*f*) is 0.40. Additionally, using parameters *α* = 0.05, *β* = 0.20, an a priori power calculation determined that each group required 20 subjects. In consideration of dropout rate, we added two more subjects to each group, for a total of 22 subjects in each group.

### 2.4 Enrollment, randomization, and blinding

We enrolled 44 of 92 subjects who were found eligible to participate in this study by the physician. We selected subjects with high sensitivity‐prostate specific antigen ≤3.0 ng/ml between the ages 40 and 64 years, or ≤ 3.5 ng/ml between the ages of 65 and 69 years; all subjects had mild‐to‐moderate symptoms (≤19 points) on IPSS and were not undergoing treatment. The number of potential subjects exceeded the number of subjects necessary for the study at the screening point. Therefore, we prioritized subjects with relatively higher scores on the IPSS. An allocation controller who was not directly involved in this study allocated subjects using Statlight #11 Version 2.10 (Yukms Co. Ltd). The allocation controller randomly assigned subjects to either the SP group (*n* = 22) or the placebo group (P group, *n* = 22) based on IPSS and age at a 1:1 ratio. Furthermore, subjects, physician, assessor of outcomes, or any others related to this study were not aware of group assignments and were not involved in the allocation. The allocation controller locked the assignment sheet until the key‐opening day.

### 2.5 Intervention

The test food capsules were made from gelatin and glycerin and included either 320 mg of SP or 0 mg of SP colored by caramel to serve as the placebo. Both capsules were determined to be identical in color, odor, and flavor by the ethics committee. All subjects were asked to consume either one SP capsule or one placebo capsule per day with approximately 200 ml of water and no chewing before going to bed for 12 weeks. The SP capsules were “Yawata saw palmetto” (YAWATA CORPORATION, Tottori, Japan). The SP was extracted by supercritical fluid (EUROMED S.A., Barcelona, the Kingdom of Spain) as previously described (Gerber et al., 2001).

### 2.6 Examination items

Supplementary Table S1 shows the study schedule. Examinations were conducted before intake of SP or P, and again 4, 8, and 12 weeks after intake. A safety evaluation was conducted before intake and at 12 weeks after intake.

#### 2.6.1 Primary outcome

Subjective symptoms related to urination were assessed using the Japanese version of IPSS (Barry et al., 1992). Seven items related to BPH on the IPSS were assessed on a scale of 0–5 points, calculating the total score. A lower score indicates better conditions.

#### 2.6.2 Secondary outcomes

Quality of life scores on the IPSS were assessed using a scale of 0–6 points. A lower score indicates a better quality of life.

Subjective symptoms related to urination status were assessed by the Japanese version of the King's Health Questionnaire (KHQ) (Kelleher, Cardozo, Khullar, & Salvatore, 1997). A total of 21 questions on the KHQ were assessed using a scale of 1–4 points or 1–5 points and classified into nine categories: general health, incontinence impact, role limitations, physical limitations, social limitations, personal limitations, emotional problems, sleep/energy disturbance, and severity measures. The incontinence impact was calculated from the question “how much do you think issues with urination affects your life” in the Japanese version of KHQ (literal translation). The total score and four items of subjective symptoms on the overactive bladder symptom score (OABSS) (Homma et al., 2006) assessed the severity of OAB. A lower score on the OABSS and KHQ indicates better conditions. The visual analog scale (VAS) assessed subjective symptoms related to sleep. “Awaking refreshed” was evaluated on a 100 mm horizontal line, with a range of “the best feeling of awaking (0 mm)” to “the worst feeling of awaking (100 mm)” on the VAS. Furthermore, “enough sleep” was evaluated by the VAS with a range of “the best sleep (0 mm)” to “the worst sleep (100 mm).” Subjects were instructed to draw a vertical line across the horizontal line at the point which most accurately described their conditions. Urinary frequency and nocturia were recorded in a daily report starting 1 week before intake. Then, the averages of urinary frequency and nocturia for each week over the course of the study were calculated. Moreover, subjects were asked to fill out the medical questionnaire regarding urinary time. We calculated the average of urinary time in the past week.

#### 2.6.3 Safety evaluation

We conducted a physical examination, urinalysis, and a blood test before intake and 12 weeks after intake. The examination items are shown in Supplementary Tables S2–S4.

#### 2.6.4 Statistical analysis

All outcomes were assessed before intake, and at 4, 8, and 12 weeks after intake. Defining “before intake” as the baseline, each assessment point was subtracted from baseline values to report the change in score (Δ4, Δ8, and Δ12 weeks).

Subject background and demographic data were aggregated based on age and physical characteristics and compared with P group using Student's *t* test. The primary and secondary outcome data in baseline and changes were expressed as the mean and standard deviation and were analyzed using the Student's *t* test at baseline. To determine the group by time response using two‐way repeated measures ANOVA, interactions between group and assessment time points were calculated and compared between groups with post hoc analysis at each assessment point when appropriate. Physical examination and blood analysis results were expressed as mean and standard deviation and analyzed using Student's *t* test at baseline and ANCOVA at 12 weeks after intake. When the ANCOVA was used for data analyses, the baseline value was used as covariates. Furthermore, urinalysis data were set to a code, where 1 was defined as “within the normal range,” and 0 was defined as “outside the normal range”; these differences were analyzed between groups using the Chi‐squared test.

All statistical analyses were two‐sided, and the significance level was set at *p* < .05 with no adjustment for multiple comparisons. All statistical analyses were performed using the Windows SPSS Version 23.0 (IBM Japan, Ltd).

## 3 RESULTS

### 3.1 Analysis set

**Figure 1:**
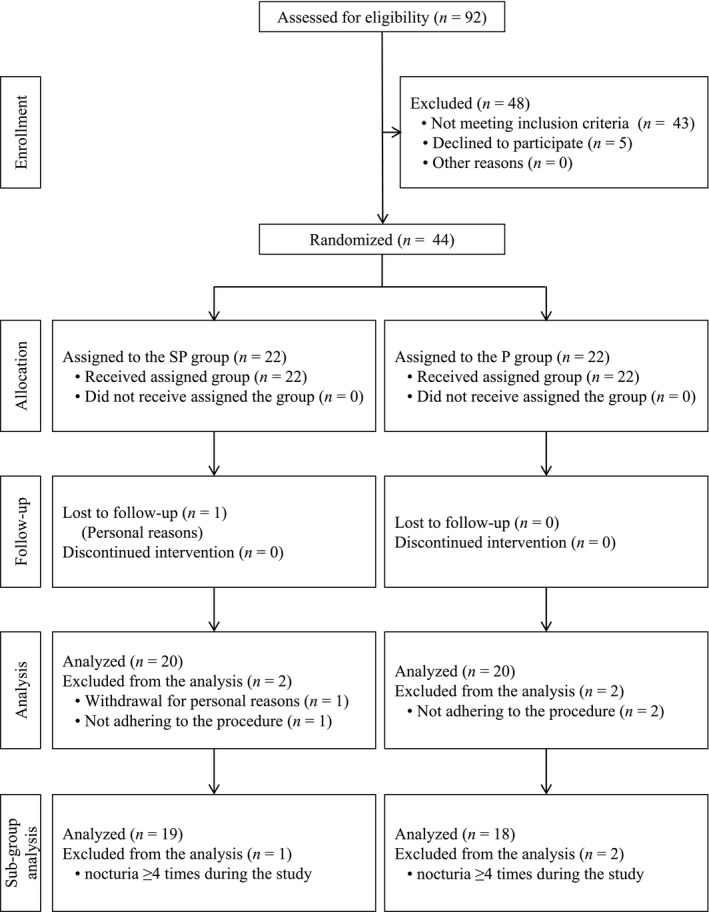
Flowchart of participant analysis in this study

Figure 1 displays the study flowchart and subject disposition. Four subjects were excluded from the analysis because one subject had lost data from missed follow‐up, and three subjects violated compliance rules. Hence, subjects were analyzed per‐protocol set with 20 subjects in the SP group and 20 subjects in the P group. Table 1 summarizes the subject characteristics in this study. Furthermore, subjects who awaken ≥4 times at night to urinate were excluded based on our results in the subgroup analysis. The subgroup analysis subjects included 19 subjects (52.4 ± 6.3 years) in the SP group and 18 (53.0 ± 7.1 years) in the P group. No significant difference was observed in their background (data not shown).

**Table 1:** Subject characteristics in SP group (*n* = 20) and P group (*n* = 20)

	SP group	P group	*p* value
Age (years)	52.3 ± 6.2	52.8 ± 6.9	.79
Body height (cm)	172.7 ± 5.5	168.9 ± 5.8	.039*
Body weight (kg)	71.0 ± 11.4	70.8 ± 10.3	.96
BMI (kg/m^2^)	23.8 ± 3.4	24.8 ± 3.2	.33
Body fat percentage (%)	20.7 ± 5.4	22.3 ± 4.4	.30
Systolic blood pressure (mmHg)	126.2 ± 14.1	123.3 ± 18.0	.57
Diastolic blood pressure (mmHg)	81.1 ± 10.2	78.4 ± 13.3	.47
Pulse rate (bpm)	68.4 ± 7.6	76.3 ± 15.6	.050
IPSS (points)	12.0 ± 4.8	10.7 ± 4.7	.38

Note

Data are presented as means ± standard deviation and were analyzed by Student's *t* test.

Abbreviations: BMI, body mass index; IPSS, international prostate symptom score.

* *p* < .05 versus the P group.

### 3.2 IPSS, KHQ, and OABSS

Table 2 presents the results of the IPSS, KHQ, and OABSS in the SP and P groups. The average change of IPSS in the SP group was low since 8 weeks, and a significant group–time interaction was observed (*p* = .047). Furthermore, the SP group showed significantly decreased IPSS compared with the P group at 8 weeks in post hoc analysis (*p* = .028).

**Table 2:** The results of variable on the IPSS, KHQ, and OABSS in SP group (*n* = 20) and P group (*n* = 20)

	Group	Screening (Baseline)	Δ4 weeks	Δ8 weeks	Δ12 weeks	Group–Time Interaction	Post hoc analysis		
							Δ4 weeks	Δ8 weeks	Δ12 weeks
IPSS									
Total IPSS (points)	SP group	12.0 ± 4.8	−1.3 ± 4.5	−4.1 ± 2.8	−4.3 ± 3.0	.047*	.93	.028*	.41
	P group	10.7 ± 4.7	−1.4 ± 3.0	−1.6 ± 4.1	−3.3 ± 4.4				
QOL (points)	SP group	4.7 ± 1.0	−0.7 ± 1.4	−0.9 ± 1.4	−1.2 ± 1.2	.22	.44	.91	.41
	P group	4.7 ± 1.1	−0.4 ± 0.9	−1.0 ± 1.3	−1.6 ± 1.4				
KHQ									
General health perception (points)	SP group	27.5 ± 18.0	−6.3 ± 16.0	−2.5 ± 16.0	1.3 ± 12.8	.52	.25	.36	1.00
	P group	22.5 ± 13.8	0.0 ± 18.1	2.5 ± 18.0	1.3 ± 15.1				
Incontinence impact (points)	SP group	46.7 ± 22.7	−10.0 ± 19.0	−6.7 ± 17.4	−18.3 ± 20.2	.013*	1.00	.27	.078
	P group	38.3 ± 22.4	−10.0 ± 21.9	−13.3 ± 19.9	−6.7 ± 20.5				
Role limitations (points)	SP group	17.5 ± 13.8	4.2 ± 17.0	3.3 ± 14.9	−1.7 ± 9.2	.39	.48	.031*	.098
	P group	22.5 ± 18.2	0.0 ± 20.2	−7.5 ± 15.7	−9.2 ± 17.5				
Physical limitations (points)	SP group	21.7 ± 17.2	−0.8 ± 15.7	0.0 ± 16.2	−3.3 ± 12.8	.062	.40	.26	.53
	P group	21.7 ± 18.8	3.3 ± 14.9	−5.0 ± 10.9	−5.8 ± 12.4				
Social limitations (points)	SP group	14.2 ± 17.0	−5.0 ± 13.4	−5.8 ± 11.9	−6.4 ± 15.8	.41	.55	.95	.72
	P group	14.4 ± 19.1	−2.2 ± 16.0	−5.6 ± 14.6	−8.3 ± 18.0				
Personal limitations (points)	SP group	7.0 ± 11.3	−0.9 ± 9.0	−1.9 ± 12.6	0.9 ± 14.1	.030*	.27	.70	.037*
	P group	14.2 ± 18.9	3.5 ± 14.2	−3.5 ± 13.1	−9.6 ± 15.0				
Emotional problems (points)	SP group	26.1 ± 19.5	−0.6 ± 15.1	0.6 ± 16.7	−6.1 ± 17.1	.26	.47	.087	.020*
	P group	30.6 ± 23.6	−5.0 ± 22.6	−8.3 ± 15.2	−18.9 ± 16.2				
Sleep/energy disturbance (points)	SP group	40.0 ± 25.6	−6.7 ± 12.6	−9.2 ± 14.8	−15.0 ± 17.0	.090	.050	.62	.48
	P group	35.0 ± 24.1	6.7 ± 26.7	−5.8 ± 25.5	−10.8 ± 19.7				
Severity measures (points)	SP group	16.7 ± 19.4	−1.3 ± 9.3	0.0 ± 9.9	−2.0 ± 8.9	.005**	.63	.16	.041*
	P group	17.3 ± 14.1	0.3 ± 12.3	−5.3 ± 13.4	−9.3 ± 12.7				
OABSS									
Total OABSS (points)	SP group	4.8 ± 1.9	−0.6 ± 1.2	−1.3 ± 1.2	−1.3 ± 1.4	.38	.42	.91	.25
	P group	4.6 ± 1.9	−1.0 ± 1.9	−1.3 ± 1.5	−1.9 ± 1.8				
Question A (points)	SP group	0.8 ± 0.6	0.1 ± 0.4	0.2 ± 0.4	0.2 ± 0.4	.78	.42	.29	.17
	P group	0.6 ± 0.6	−0.1 ± 0.7	0.0 ± 0.7	−0.1 ± 0.7				
Question B (points)	SP group	2.1 ± 0.3	−0.6 ± 0.6	−0.9 ± 0.6	−0.9 ± 0.5	.074	.47	.64	.17
	P group	2.2 ± 0.4	−0.5 ± 0.7	−0.8 ± 0.7	−1.1 ± 0.6				
Question C (points)	SP group	1.7 ± 1.3	−0.1 ± 1.0	−0.6 ± 1.0	−0.7 ± 1.0	.19	.39	.85	.76
	P group	1.6 ± 1.2	−0.4 ± 1.1	−0.5 ± 0.7	−0.6 ± 1.1				
Question D (points)	SP group	0.3 ± 0.6	0.1 ± 0.2	0.0 ± 0.7	0.1 ± 0.5	.42	.43	1.00	.21
	P group	0.3 ± 0.5	−0.1 ± 0.5	0.0 ± 0.6	−0.2 ± 0.5				

Note

The data are presented as the mean ± standard deviation and were analyzed by repeated measures two‐way ANOVA. Question A, how many times do you typically urinate from waking in the morning until sleeping at night? Question B, how many times do you typically wake up to urinate from sleeping at night until waking in the morning? Question C, how often do you have a sudden desire to urinate, which is difficult to defer? Question D, how often do you leak urine because you cannot defer the sudden desire to urinate.

Abbreviations: IPSS, international prostate symptom score; KHQ, King's health questionnaire; OABSS, overactive bladder symptom score.

* *p* < .05.

** *p* < .01 versus the P group.

A significant group–time interaction of incontinence impact of KHQ scores changes was observed (*p* = .013), and the SP group tended to have lower change in incontinence impact of KHQ scores than in the P group at 12 weeks in post hoc analysis (*p* = .078). The subgroup analysis results are shown in Table 3. The average change of IPSS in the SP group was lower at change over time throughout the intervention period, and a significant group–time interaction was observed (*p* = .044). Furthermore, the SP group showed significantly decreased IPSS compared with the P group at 8 weeks in post hoc analysis (*p* = .040). A significant group–time interaction of incontinence impact of KHQ scores changes was observed (*p* = .014), and the SP group had significantly lower change in incontinence impact of KHQ than the P group at 12 weeks in post hoc analysis (*p* = .042). There were no significant differences in OABSS between groups.

**Table 3:** The results of variable on the IPSS, KHQ, and OABSS in SP group (*n* = 19) and P group (*n* = 18)

	Group	Screening (Baseline)	Δ4 weeks	Δ8 weeks	Δ12 weeks	Group–Time Interaction	Post hoc analysis		
							Δ4 weeks	Δ8 weeks	Δ12 weeks
IPSS									
Total IPSS (points)	SP group	12.1 ± 5.0	−1.7 ± 4.2	−3.9 ± 2.7	−4.2 ± 3.1	.044*	.84	.040*	.29
	P group	10.0 ± 4.5	−1.4 ± 2.9	−1.4 ± 4.3	−2.8 ± 4.3				
QOL (points)	SP group	4.6 ± 1.0	−0.6 ± 1.3	−0.7 ± 1.2	−1.1 ± 1.1	.25	.73	.44	.26
	P group	4.5 ± 1.0	−0.4 ± 1.0	−1.1 ± 1.3	−1.5 ± 1.3				
KHQ									
General health perception (points)	SP group	27.6 ± 18.4	−6.6 ± 16.3	−1.3 ± 15.5	1.3 ± 13.1	.48	.24	.81	.99
	P group	20.8 ± 12.9	0.0 ± 17.1	0.0 ± 17.1	1.4 ± 13.5				
Incontinence impact (points)	SP group	47.4 ± 23.1	−8.8 ± 18.7	−5.3 ± 16.7	−17.5 ± 20.4	.014*	.94	.45	.042*
	P group	33.3 ± 16.2	−9.3 ± 22.3	−9.3 ± 15.4	−3.7 ± 19.4				
Role limitations (points)	SP group	18.4 ± 13.5	4.4 ± 17.4	3.5 ± 15.3	−1.8 ± 9.5	.54	.50	.055	.16
	P group	19.4 ± 15.4	0.0 ± 21.4	−6.5 ± 15.3	−8.3 ± 17.4				
Physical limitations (points)	SP group	22.8 ± 16.9	−0.9 ± 16.2	0.0 ± 16.7	−3.5 ± 13.1	.096	.39	.41	.60
	P group	18.5 ± 16.1	3.7 ± 15.7	−3.7 ± 9.1	−5.6 ± 9.9				
Social limitations (points)	SP group	14.9 ± 17.2	−5.3 ± 13.7	−6.1 ± 12.1	−6.7 ± 16.2	.70	.33	.57	.71
	P group	11.7 ± 15.5	−0.6 ± 15.0	−3.7 ± 13.7	−4.9 ± 12.2				
Personal limitations (points)	SP group	7.4 ± 11.5	−1.0 ± 9.3	−2.0 ± 13.0	0.9 ± 14.5	.063	.26	1.00	.07
	P group	12.0 ± 14.9	3.9 ± 15.1	−2.0 ± 11.6	−7.8 ± 12.0				
Emotional problems (points)	SP group	27.5 ± 19.0	−0.6 ± 15.5	0.6 ± 17.2	−6.4 ± 17.5	.61	.40	.079	.073
	P group	27.2 ± 22.3	−6.2 ± 23.6	−9.3 ± 15.8	−16.0 ± 13.9				
Sleep/energy disturbance (points)	SP group	41.2 ± 25.7	−6.1 ± 12.7	−8.8 ± 15.1	−14.9 ± 17.5	.22	.16	.94	.62
	P group	33.3 ± 24.3	2.8 ± 23.7	−8.3 ± 19.2	−12.0 ± 17.0				
Severity measures (points)	SP group	17.5 ± 19.5	−1.4 ± 9.6	0.0 ± 10.2	−2.1 ± 9.2	.008**	.56	.17	.076
	P group	15.2 ± 12.7	0.7 ± 12.5	−5.6 ± 13.8	−8.9 ± 13.1				
OABSS									
Total OABSS (points)	SP group	4.9 ± 1.9	−0.6 ± 1.2	−1.2 ± 1.2	−1.3 ± 1.4	.50	.21	.60	.20
	P group	4.4 ± 1.7	−1.2 ± 1.8	−1.4 ± 1.5	−2.0 ± 1.8				
Question A (points)	SP group	0.8 ± 0.5	0.1 ± 0.4	0.2 ± 0.4	0.2 ± 0.4	.91	.41	.43	.26
	P group	0.6 ± 0.6	−0.1 ± 0.8	0.1 ± 0.7	−0.1 ± 0.7				
Question B (points)	SP group	2.1 ± 0.2	−0.5 ± 0.5	−0.8 ± 0.6	−0.8 ± 0.5	.24	.89	.96	.14
	P group	2.1 ± 0.3	−0.5 ± 0.6	−0.8 ± 0.5	−1.1 ± 0.6				
Question C (points)	SP group	1.7 ± 1.3	−0.2 ± 1.0	−0.6 ± 1.0	−0.7 ± 1.1	.25	.34	.94	.96
	P group	1.5 ± 1.1	−0.5 ± 1.2	−0.6 ± 0.7	−0.7 ± 1.0				
Question D (points)	SP group	0.3 ± 0.6	0.1 ± 0.2	0.0 ± 0.7	0.1 ± 0.5	.72	.18	.59	.21
	P group	0.3 ± 0.5	−0.1 ± 0.5	−0.1 ± 0.5	−0.2 ± 0.5				

Note

The data are presented as the means ± standard deviation and were analyzed by repeated measures two‐way ANOVA. Question A, how many times do you typically urinate from waking in the morning until sleeping at night? Question B, how many times do you typically wake up to urinate from sleeping at night until waking in the morning? Question C, how often do you have a sudden desire to urinate, which is difficult to defer? Question D, how often do you leak urine because you cannot defer the sudden desire to urinate?

Abbreviations: IPSS, international prostate symptom score; KHQ, King's health questionnaire; OABSS, overactive bladder symptom score.

* *p* < .05.

** *p* < .01 versus the P group.

### 3.3 VAS related to sleep, time of urination, and daily report

There were no significant differences in VAS regarding sleep, time of urination, and daily report between groups (data not shown).

### 3.4 Physical examination, urinalysis, and blood tests

We observed no medically problematic changes in either group throughout the study period (Supplementary Tables S2–S4). Although some subjects had blood pressure outside the optimal range (The Japanese Society of Hypertension Committee for Guidelines for the Management of Hypertension 2014), we judged including other examinations that the subjects were healthy at screening and examination at the hospital visit.

## 4 DISCUSSION

A significant group–time interaction for IPSS changes was observed. Furthermore, IPSS was significantly decreased in the SP group compared with the P group at 8 weeks in post hoc analysis. The lower score indicates urination subjective symptoms were alleviated with intake of the SP capsule. IPSS, the primary outcome of this study, is also used in the other studies performed with healthy Japanese men (Kushima et al., 2018; Najima et al., 2017; Sekikawa, Kizawa, Li, & Takara, 2020). In all those studies, consumption of the test food significantly reduced the IPSS, and the authors concluded that the test foods alleviated the urination subjective symptoms (Kushima et al., 2018; Najima et al., 2017; Sekikawa et al., 2020). Thus, the significant reduction of IPSS in the SP group of this study can be interpreted as the change of urination subjective symptoms. Therefore, IPSS is an index that can evaluate the change of urination subjective symptoms of healthy Japanese subjects, and it is considered that the effect of the SP could be appropriately detected in this study.

Lower urinary tract symptoms is caused by two types of obstructions including functional obstruction, in which the prostate smooth muscle contracts to narrow the urethra, and mechanical obstruction, in which the prostate gland physically hinders urination (Kawabe, 2006). The functional obstruction is caused by muscle hypertonia due to α_1_‐adrenergic receptor stimulation of the prostatic smooth muscle (Schwinn & Roehrborn, 2008). In a previous study in rats, SP inhibits the α_1_‐adrenergic receptor and muscarinic acetylcholine receptor, contributing to an increase in bladder capacity and prolongation of the micturition desire interval (Oki et al., 2005). On the contrary, suppression of BPH is necessary to improve the mechanical obstruction. Testosterone reacts with 5α‐reductase to produce dihydrotestosterone (DHT), leading to the development of BPH (Bartsch, Rittmaster, & Klocker, 2002). Fatty acids, including lauric acid, oleic acid, myristic acid, and linoleic acid (the main components of SP), functionally inhibit the activity of 5α‐reductase (Abe, Ito, Oyunzul, Oki‐Fujino, & Yamada, 2009; Abe, Ito, Suzuki, et al., 2009); thus, SP is confirmed to suppress DHT production (Habib, Ross, Ho, Lyons, & Chapman, 2005). Furthermore, SP inhibits binding of androgen receptor as a target of DHT and also promoted apoptosis of the prostatic epithelium; these effects also suppress prostate enlargement (Suzuki et al., 2009; Vela‐Navarrete et al., 2005). β‐Sitosterols in SP are effective against BPH associated with urinary symptoms and improved urine flow in humans (Wilt et al., 1999). Additionally, free fatty acids in SP influence physiological functions related to urination such as permeability of ion channels (K^+^, Na^+^, and Ca^2+^) and neurotransmission; therefore, free fatty acids directly affect the lower urinary tract nerve (Ordway, Singer, & Walsh, 1991). In a previous study in rats, fatty acids, such as lauric acid, oleic acid, and β‐Sitosterol, were shown to be absorbed more in prostatic glandular tissues than in other tissues (Chevalier, Benard, Cousse, & Bengone, 1997; Pérez, Menéndez, Más, & González, 2006). Thus, the consumption of SP alleviated subjective symptoms related to frequent urination via a complex interaction with prostate smooth muscle relaxation, prostate enlargement inhibition, and lower urinary tract nerve.

The SP group had significantly improved incontinence impact as assessed by the KHQ. Because a lower KHQ score indicates a better condition, intake of SP may improve urination issues and thus improve daily living. However, because the KHQ score did not continuously show low scores, the effects of SP on incontinence impact were limited. In addition, significant group–time interactions were observed in personal limitations and severity measures; however, both items in post hoc analysis showed significantly lower scores in P group than in SP group. The score of personal limitation is calculated from questions about sexual life; there were few subjects who had sexual problems according to the results of KHQ. Thus, the difference in KHQ score of personal limitation was not considered to be clinically important. Furthermore, subjects in this study were not currently on any medications or treatment because of urinary disorders, such as OAB, and most of them did not suffer from severe subjective symptoms, as those mentioned in the questionnaire. Therefore, we concluded that the statistically significant difference observed in severity measures was not clinically meaningful.

We observed no significant differences between groups in the OABSS. The diagnostic criteria for OAB are a total score of ≥ 3 points and ≥ 2 points on question C (urinary urgency score) on the OABSS (Yamaguchi et al., 2009). Subjects in this study were healthy volunteers who were not currently on any medications or treatment due to disorders including OAB; the total score was ≥3 points, but question C was <2 points from screening throughout the intervention period. No subjects in this study were diagnosed with OAB; therefore, there were no significant changes in the score. In this study, the P group also showed a reduction in subjective symptoms related to urination issues with the significant group–time interaction; however, the improvement in scores was inconsistent.

The subgroup analysis showed an improvement in items in IPSS like per‐protocol analysis in this study. The average change of IPSS in the SP group was low throughout the intervention period, and a significant group–time interaction was observed. Further, IPSS of the SP group significantly decreased than that of the P group at 8 weeks in post hoc analysis. The effect of SP on incontinence impact of KHQ was restrictive similar to the main per‐protocol analysis. Therefore, SP capsules could have effect even in those with relatively mild symptoms (who awaken <4 times at night to urinate).

The results of our study are consistent with the effects reported by Gerber et al. (2001). Symptoms for urination issues improved, and this might be confirmed by objective evaluations. Future studies should include the evaluation of urodynamics and uroflowmetry tests (Yachiku, 1981).

## 5 CONCLUSIONS

The consumption of saw palmetto fruit extract capsules for 12 weeks relieved urination‐related subjective symptoms, which suggests improved issues in Japanese men of 40–69 years of age who experienced urination issues, such as urinary urgency, frequent urination, urinary incontinence, and awaken ≥2 times at night to urinate. Furthermore, we determined that the consumption of SP capsules was safe under the conditions of this study.

## CONFLICTS OF INTEREST

I. I. is affiliated with YAWATA CORPORATION and T. W. is part of NIHON PHARMACEUTICAL CO., LTD. Both companies were sponsors of the study, and supported the study by providing expenses and fees for the experiment and subsequent drafting of the manuscript. The YAWATA CORPORATION and NIHON PHARMACEUTICAL CO., LTD. entrusted ORTHOMEDICO Inc. with conducting the study in collaboration. T. T. (MD) belongs to the Medical Corporation Seishinkai, Takara Clinic, who was the principal investigator and managed the physical condition of all subjects at his clinic.

## ETHICS STATEMENT

This study conforms to the Declaration of Helsinki (2013). All protocols and procedures were ethically reviewed and approved by the independent ethical committee of the clinic set up by Medical Corporation Seishinkai on June 19, 2017 (Approval ID: 1706–1704‐YB02‐01‐TC). Written and verbal informed consent was collected from all subjects prior to participation.

## Supporting information

Table S1‐S4
