# Supplementary material for: Effects of saw palmetto fruit extract intake on improving urination issues in Japanese men: A randomized, double‐blind, parallel‐group, placebo‐controlled study
Source: Food Sci Nutr. 2020 Jun 17;8(8):4017–26. doi: 10.1002/fsn3.1654 (PMC7455929; doi:10.1002/fsn3.1654)
Supplement: Supplementary file 1 — Table S1‐S4 [file FSN3-8-4017-s001.docx]

| Supplementary Table S1. Schedule of enrollment, intervention, and assessments | | | | | | | | | | |
| --- | --- | --- | --- | --- | --- | --- | --- | --- | --- | --- |
| **TIME POINT** | **STUDY PERIOD** | | | | | | | | | |
|  | **Enrollment** |  | **Screening (Baseline)** |  | **Allocation** |  | **Start intake** | **Post-allocation** | | |
|  |  |  |  |  |  |  |  | **4 weeks  after intake** | **8 weeks  after intake** | **12 weeks  after intake** |
| ENROLLMENT: |  |  |  |  |  |  |  |  |  |  |
| Eligibility screen | × |  |  |  |  |  |  |  |  |  |
| Informed consent | × |  |  |  |  |  |  |  |  |  |
| Allocation |  |  |  |  | × |  |  |  |  |  |
| INTERVENTIONS: |  |  |  |  |  |  |  |  |  |  |
| SP group |  |  |  |  |  |  |  | | | |
| P group |  |  |  |  |  |  |  | | | |
| ASSESSMENTS: |  |  |  |  |  |  |  |  |  |  |
| IPSS |  |  | × |  |  |  |  | × | × | × |
| KHQ |  |  | × |  |  |  |  | × | × | × |
| OABSS |  |  | × |  |  |  |  | × | × | × |
| Physical examination |  |  | × |  |  |  |  | × | × | × |
| Urinalysis |  |  | × |  |  |  |  | × | × | × |
| Hematological and blood biochemical tests |  |  | × |  |  |  |  | × | × | × |
| Daily record |  |  |  |  |  |  |  | | | |
| Medical questionnaire |  |  | × |  |  |  |  | × | × | × |

IPSS, international prostate symptom score; KHQ, King's health questionnaire; OABSS, overactive bladder symptom score

| Supplementary Table S2. The results of physical examination in SP group (*n* = 20) and P group (*n* = 20) | | | | | | | | | |
| --- | --- | --- | --- | --- | --- | --- | --- | --- | --- |
|  | **Group** | **Screening** | | | **12 weeks** | | | ***P* value** | |
|  |  |  |  |  |  |  |  | **Screening** | **12 weeks** |
| Body height (cm) | SP group | 172.7 | ± | 5.5 | ― | | | .039* | ― |
|  | P group | 168.9 | ± | 5.8 | ― | | |  |  |
| Body weight (kg) | SP group | 71.0 | ± | 11.4 | 72.0 | ± | 11.9 | .96 | .90 |
|  | P group | 70.8 | ± | 10.3 | 71.7 | ± | 10.6 |  |  |
| BMI (kg/m^2^) | SP group | 23.8 | ± | 3.4 | 24.1 | ± | 3.6 | .33 | .90 |
|  | P group | 24.8 | ± | 3.2 | 25.1 | ± | 3.3 |  |  |
| Body fat percentage (%) | SP group | 20.7 | ± | 5.4 | 19.6 | ± | 6.2 | .30 | .26 |
|  | P group | 22.3 | ± | 4.4 | 22.1 | ± | 4.6 |  |  |
| Systolic blood pressure (mmHg) | SP group | 126.2 | ± | 14.1 | 138.7 | ± | 16.7 | .57 | .024* |
|  | P group | 123.3 | ± | 18.0 | 128.1 | ± | 19.8 |  |  |
| Diastolic blood pressure (mmHg) | SP group | 81.1 | ± | 10.2 | 89.0 | ± | 11.9 | .47 | .038* |
|  | P group | 78.4 | ± | 13.3 | 82.1 | ± | 11.5 |  |  |
| Pulse rate (bpm) | SP group | 68.4 | ± | 7.6 | 67.1 | ± | 6.8 | .050 | .52 |
|  | P group | 76.3 | ± | 15.6 | 72.9 | ± | 11.5 |  |  |

The data are presented as the means ± standard deviation and were analyzed by ANCOVA

**P* < .05 vs. the P group

BMI, body mass index

| Supplementary Table S3. The results of urinalysis in SP group (*n* = 20) and P group (*n* = 20) | | | | | | | |
| --- | --- | --- | --- | --- | --- | --- | --- |
|  | **Assessment points** | **SP group** | |  | **P group** | | ***P* value** |
|  |  | **Within the reference values** | **Outside of the reference values** |  | **Within the reference values** | **Outside of the reference values** |  |
| Protein | Screening | 15 | 5 |  | 18 | 2 | .41 |
|  | 12 weeks | 15 | 5 |  | 15 | 5 | 1.00 |
| Glucose | Screening | 20 | 0 |  | 18 | 2 | .49 |
|  | 12 weeks | 20 | 0 |  | 20 | 0 | N.A. |
| Urobilinogen | Screening | 20 | 0 |  | 20 | 0 | N.A. |
|  | 12 weeks | 20 | 0 |  | 20 | 0 | N.A. |
| Bilirubin | Screening | 20 | 0 |  | 20 | 0 | N.A. |
|  | 12 weeks | 20 | 0 |  | 20 | 0 | N.A. |
| pH | Screening | 19 | 1 |  | 20 | 0 | 1.00 |
|  | 12 weeks | 20 | 0 |  | 20 | 0 | N.A. |
| Occult blood | Screening | 18 | 2 |  | 19 | 1 | 1.00 |
|  | 12 weeks | 17 | 3 |  | 18 | 2 | 1.00 |
| Ketone bodies | Screening | 20 | 0 |  | 20 | 0 | N.A. |
|  | 12 weeks | 20 | 0 |  | 20 | 0 | N.A. |

The data are presented as number of participants and were analyzed using the Chi-squared test

N.A., Not available

| Supplementary Table S4. The results of blood test in SP group (*n* = 20) and P group (*n* = 20) | | | | | | | | | | |
| --- | --- | --- | --- | --- | --- | --- | --- | --- | --- | --- |
|  | **Reference value** | **Group** | **Screening** | | | **12 weeks** | | | ***P* value** | |
|  |  |  |  |  |  |  |  |  | **Screening** | **12 weeks** |
| Leukocyte count (/μL) | 3300–9000 | SP group | 5330.0 | ± | 1422.0 | 5660.0 | ± | 1912.7 | .12 | .87 |
|  |  | P group | 5955.0 | ± | 1021.6 | 5880.0 | ± | 1285.8 |  |  |
| Erythrocyte count (×10^4^/μL) | 430–570 | SP group | 483.7 | ± | 26.3 | 481.4 | ± | 26.0 | .33 | .14 |
|  |  | P group | 474.1 | ± | 35.1 | 481.7 | ± | 38.4 |  |  |
| Hemoglobin (g/dL) | 13.5–17.5 | SP group | 15.1 | ± | 0.8 | 15.1 | ± | 0.8 | .31 | .25 |
|  |  | P group | 14.8 | ± | 1.0 | 15.0 | ± | 1.2 |  |  |
| Hematocrit value (%) | 39.7–52.4 | SP group | 46.4 | ± | 2.0 | 46.2 | ± | 2.0 | .10 | .015* |
|  |  | P group | 45.1 | ± | 2.6 | 46.5 | ± | 3.4 |  |  |
| Platelet count (×10^4^/μL) | 14.0–34.0 | SP group | 26.5 | ± | 4.7 | 26.3 | ± | 4.8 | .85 | .22 |
|  |  | P group | 26.2 | ± | 6.4 | 26.7 | ± | 7.4 |  |  |
| Mean corpuscular volume (fL) | 85–102 | SP group | 95.9 | ± | 3.6 | 96.2 | ± | 3.9 | .64 | .16 |
|  |  | P group | 95.3 | ± | 5.1 | 96.8 | ± | 5.0 |  |  |
| Mean corpuscular hemoglobin (pg) | 28.0–34.0 | SP group | 31.2 | ± | 1.3 | 31.3 | ± | 1.2 | .95 | .59 |
|  |  | P group | 31.2 | ± | 1.8 | 31.2 | ± | 1.8 |  |  |
| Mean corpuscular hemoglobin concentration (%) | 30.2–35.1 | SP group | 32.5 | ± | 0.9 | 32.6 | ± | 0.7 | .35 | .07 |
|  |  | P group | 32.7 | ± | 0.7 | 32.3 | ± | 0.7 |  |  |
| Percentage of neutrophils (%) | 40.0–75.0 | SP group | 59.2 | ± | 8.4 | 61.5 | ± | 8.7 | .32 | .39 |
|  |  | P group | 56.8 | ± | 6.7 | 58.6 | ± | 5.9 |  |  |
| Percentage of lymphocytes (%) | 18.0–49.0 | SP group | 32.1 | ± | 8.2 | 29.4 | ± | 8.2 | .28 | .41 |
|  |  | P group | 34.7 | ± | 6.5 | 32.2 | ± | 5.5 |  |  |
| Percentage of monocytes (%) | 2.0–10.0 | SP group | 5.3 | ± | 1.3 | 5.7 | ± | 1.4 | .99 | .52 |
|  |  | P group | 5.3 | ± | 1.4 | 5.5 | ± | 1.4 |  |  |
| Percentage of eosinophils (%) | 0.0–8.0 | SP group | 2.7 | ± | 1.3 | 2.7 | ± | 1.7 | .71 | .40 |
|  |  | P group | 2.5 | ± | 1.6 | 2.9 | ± | 1.7 |  |  |
| Percentage of basophils (%) | 0.0–2.0 | SP group | 0.7 | ± | 0.5 | 0.7 | ± | 0.4 | .95 | .36 |
|  |  | P group | 0.7 | ± | 0.4 | 0.8 | ± | 0.5 |  |  |
| AST (U/L) | 10–40 | SP group | 22.2 | ± | 6.8 | 22.0 | ± | 5.7 | .38 | .90 |
|  |  | P group | 25.0 | ± | 12.1 | 23.5 | ± | 6.9 |  |  |
| ALT (U/L) | 5–45 | SP group | 21.3 | ± | 8.6 | 20.5 | ± | 6.9 | .62 | .45 |
|  |  | P group | 22.9 | ± | 10.6 | 22.9 | ± | 9.4 |  |  |
| γ-GT (U/L) | ≤80 | SP group | 44.8 | ± | 41.9 | 41.2 | ± | 31.0 | .54 | .89 |
|  |  | P group | 59.4 | ± | 96.0 | 52.6 | ± | 73.6 |  |  |
| ALP (U/L) | 100–325 | SP group | 207.0 | ± | 45.5 | 202.5 | ± | 40.2 | .22 | .49 |
|  |  | P group | 226.9 | ± | 54.7 | 224.5 | ± | 56.5 |  |  |
| LD (U/L) | 120–240 | SP group | 200.2 | ± | 18.7 | 196.0 | ± | 12.2 | .38 | .57 |
|  |  | P group | 209.7 | ± | 44.0 | 196.6 | ± | 22.7 |  |  |
| LAP (U/L) | 45–81 | SP group | 53.3 | ± | 10.2 | 51.6 | ± | 8.1 | .43 | .82 |
|  |  | P group | 56.3 | ± | 13.5 | 54.2 | ± | 11.9 |  |  |
| Total bilirubin (mg/dL) | 0.2–1.2 | SP group | 0.9 | ± | 0.3 | 0.9 | ± | 0.2 | .85 | .98 |
|  |  | P group | 0.9 | ± | 0.4 | 0.9 | ± | 0.4 |  |  |
| Direct bilirubin (mg/dL) | 0.0–0.2 | SP group | 0.1 | ± | 0.0 | 0.1 | ± | 0.0 | .58 | .22 |
|  |  | P group | 0.1 | ± | 0.1 | 0.1 | ± | 0.0 |  |  |
| Indirect bilirubin (mg/dL) | 0.2–1.0 | SP group | 0.8 | ± | 0.3 | 0.7 | ± | 0.2 | .91 | .86 |
|  |  | P group | 0.8 | ± | 0.3 | 0.8 | ± | 0.4 |  |  |
| Cholinesterase (U/L) | 234–493 | SP group | 350.3 | ± | 38.5 | 347.7 | ± | 48.5 | .89 | .42 |
|  |  | P group | 347.5 | ± | 78.0 | 350.9 | ± | 74.5 |  |  |
| ZTT (U) | 2.0–12.0 | SP group | 6.7 | ± | 3.5 | 6.5 | ± | 2.9 | .28 | .70 |
|  |  | P group | 5.6 | ± | 2.8 | 5.5 | ± | 2.7 |  |  |
| Total protein (g/dL) | 6.7–8.3 | SP group | 7.4 | ± | 0.3 | 7.2 | ± | 0.3 | .06 | .84 |
|  |  | P group | 7.2 | ± | 0.3 | 7.1 | ± | 0.2 |  |  |
| Urea nitrogen (mg/dL) | 8.0–20.0 | SP group | 13.0 | ± | 3.2 | 13.7 | ± | 3.9 | .021* | .93 |
|  |  | P group | 15.7 | ± | 3.9 | 16.0 | ± | 4.3 |  |  |
| Creatinine (mg/dL) | 0.61–1.04 | SP group | 0.9 | ± | 0.1 | 0.8 | ± | 0.1 | .35 | .45 |
|  |  | P group | 0.9 | ± | 0.1 | 0.8 | ± | 0.1 |  |  |
| Uric acid (mg/dL) | 3.8–7.0 | SP group | 5.8 | ± | 1.3 | 5.6 | ± | 1.1 | .65 | .76 |
|  |  | P group | 6.1 | ± | 1.9 | 5.7 | ± | 1.6 |  |  |
| CK (U/L) | 60–270 | SP group | 150.6 | ± | 58.9 | 144.1 | ± | 45.8 | .26 | .34 |
|  |  | P group | 206.4 | ± | 211.3 | 133.7 | ± | 56.8 |  |  |
| Sodium (mEq/L) | 137–147 | SP group | 142.6 | ± | 1.4 | 142.2 | ± | 1.3 | .92 | .45 |
|  |  | P group | 142.7 | ± | 1.7 | 141.9 | ± | 1.3 |  |  |
| Potassium (mEq/L) | 3.5–5.0 | SP group | 4.1 | ± | 0.2 | 4.3 | ± | 0.3 | .20 | .35 |
|  |  | P group | 3.9 | ± | 0.3 | 4.3 | ± | 0.5 |  |  |
| Chloride (mEq/L) | 98–108 | SP group | 101.6 | ± | 1.7 | 102.4 | ± | 1.6 | .52 | .13 |
|  |  | P group | 102.1 | ± | 2.6 | 101.8 | ± | 2.1 |  |  |
| Calcium (mEq/L) | 8.4–10.4 | SP group | 9.5 | ± | 0.2 | 9.4 | ± | 0.3 | .78 | .31 |
|  |  | P group | 9.5 | ± | 0.3 | 9.5 | ± | 0.2 |  |  |
| Inorganic phosphorus (mEq/L) | 2.5–4.5 | SP group | 3.6 | ± | 0.5 | 3.1 | ± | 0.4 | .11 | .12 |
|  |  | P group | 3.3 | ± | 0.7 | 2.9 | ± | 0.3 |  |  |
| Serum iron (μg/dL) | 50–200 | SP group | 120.3 | ± | 39.7 | 112.8 | ± | 33.1 | .38 | .31 |
|  |  | P group | 132.8 | ± | 48.8 | 128.7 | ± | 45.8 |  |  |
| Serum amylase (U/L) | 40–122 | SP group | 68.8 | ± | 16.9 | 68.2 | ± | 12.3 | .81 | .19 |
|  |  | P group | 70.0 | ± | 13.7 | 73.4 | ± | 17.2 |  |  |
| Total cholesterol (mg/dL) | 120–219 | SP group | 212.9 | ± | 34.2 | 209.0 | ± | 32.7 | .37 | .51 |
|  |  | P group | 204.3 | ± | 24.8 | 206.0 | ± | 32.6 |  |  |
| HDL cholesterol (mg/dL) | 40–85 | SP group | 68.1 | ± | 20.4 | 67.7 | ± | 17.7 | .20 | .53 |
|  |  | P group | 61.0 | ± | 13.9 | 60.2 | ± | 15.0 |  |  |
| LDL cholesterol (mg/dL) | 65–139 | SP group | 121.6 | ± | 30.6 | 120.1 | ± | 31.0 | .44 | .22 |
|  |  | P group | 114.6 | ± | 26.4 | 120.8 | ± | 34.1 |  |  |
| Triglyceride (mg/dL) | 30–149 | SP group | 110.0 | ± | 55.6 | 90.0 | ± | 45.0 | .30 | .13 |
|  |  | P group | 145.8 | ± | 140.0 | 130.7 | ± | 84.3 |  |  |
| Glucose (mg/dL) | 70–109 | SP group | 85.5 | ± | 7.1 | 86.5 | ± | 10.2 | .35 | .19 |
|  |  | P group | 88.1 | ± | 9.9 | 94.6 | ± | 19.2 |  |  |
| Hemoglobin A1c (%) | 4.6–6.2 | SP group | 5.4 | ± | 0.2 | 5.4 | ± | 0.2 | .36 | .98 |
|  |  | P group | 5.5 | ± | 0.3 | 5.5 | ± | 0.3 |  |  |
| Glycoalbumin (%) | 12.3–16.5 | SP group | 14.0 | ± | 1.2 | 13.8 | ± | 1.1 | .89 | .46 |
|  |  | P group | 13.9 | ± | 1.2 | 13.8 | ± | 1.1 |  |  |
| high-sensitivity prostate-specific antigen (ng/mL) | ≤4.00 | SP group | 1.2 | ± | 0.4 | 1.1 | ± | 0.4 | .26 | .80 |
|  |  | P group | 1.0 | ± | 0.4 | 1.0 | ± | 0.5 |  |  |

The data are presented as the means ± standard deviation and were analyzed by Student's *t*-test

**P* < .05; ***P* < .01 vs. the P group
